# Topical application of synthetic melanin promotes tissue repair

**DOI:** 10.1038/s41536-023-00331-1

**Published:** 2023-11-02

**Authors:** Dauren Biyashev, Zofia E. Siwicka, Ummiye V. Onay, Michael Demczuk, Dan Xu, Madison K. Ernst, Spencer T. Evans, Cuong V. Nguyen, Florencia A. Son, Navjit K. Paul, Naneki C. McCallum, Omar K. Farha, Stephen D. Miller, Nathan C. Gianneschi, Kurt Q. Lu

**Affiliations:** 1 Department of Dermatology, Feinberg School of Medicine, Northwestern University, Chicago, IL USA; 2 Department of Chemistry, Northwestern University, Evanston, IL USA; 3 International Institute of Nanotechnology, Simpson-Querrey Institute, Chemistry of Life Processes Institute, Lurie Cancer Center. Northwestern University, Evanston, IL USA; 4 Department of Microbiology-Immunology, Feinberg School of Medicine, Northwestern University, Chicago, IL USA; 5 Department of Chemical and Biological Engineering, Northwestern University, Evanston, IL USA; 6 Department of Materials Science and Engineering, Northwestern University, Evanston, IL USA; 7 Department of Biomedical Engineering, Northwestern University, Evanston, IL USA; 8 Department of Pharmacology, Feinberg School of Medicine, Northwestern University, Chicago, IL USA; 9 Department of Chemistry, University of California San Diego, San Diego, Ca USA

**Keywords:** Biomaterials, Trauma, Cell death and immune response

## Abstract

In acute skin injury, healing is impaired by the excessive release of reactive oxygen species (ROS). Melanin, an efficient scavenger of radical species in the skin, performs a key role in ROS scavenging in response to UV radiation and is upregulated in response to toxic insult. In a chemical injury model in mice, we demonstrate that the topical application of synthetic melanin particles (SMPs) significantly decreases edema, reduces eschar detachment time, and increases the rate of wound area reduction compared to vehicle controls. Furthermore, these results were replicated in a UV-injury model. Immune array analysis shows downregulated gene expression in apoptotic and inflammatory signaling pathways consistent with histological reduction in apoptosis. Mechanistically, synthetic melanin intervention increases superoxide dismutase (SOD) activity, decreases *Mmp9* expression, and suppresses ERK1/2 phosphorylation. Furthermore, we observed that the application of SMPs caused increased populations of anti-inflammatory immune cells to accumulate in the skin, mirroring their decrease from splenic populations. To enhance antioxidant capacity, an engineered biomimetic High Surface Area SMP was deployed, exhibiting increased wound healing efficiency. Finally, in human skin explants, SMP intervention significantly decreased the damage caused by chemical injury. Therefore, SMPs are promising and effective candidates as topical therapies for accelerated wound healing, including via pathways validated in human skin.

## Introduction

Skin is the organ most exposed to external environmental damaging factors. These factors include damage from UV-light exposure, traumas and chronic wounds combined with perturbance during medical procedures. With these insults in mind, it becomes imperative to develop new therapies and to keep improving approaches that are currently available^1,2^. Skin wound healing is a complex process requiring a coordinated interplay of multiple cell types, growth factors and signaling molecules and our understanding of these processes will lead to advancements in how we accelerate and influence healing. Indeed, recently, significant advancements have been made in our understanding of wound repair mechanisms including the role of monocytes and macrophages^3,4^, the differentiation of fibroblasts and the functions of myofibroblasts^5^, as well as the coordination between the immune system and tissue stem cells^6,7^.

Oxidative stress is one of the main mechanisms underlying impaired healing in skin injury and in chronic wounds^8,9^. While low levels of reactive oxygen species (ROS) serve essential antimicrobial and signaling roles in wound healing^10–13^, excessive production of free radical species overwhelms the delicate oxidant-antioxidant homeostatic balance and leads to molecular dysfunction, cellular damage, and pathologic inflammation. This makes the redox system a natural target for wound-healing therapy^14,15^. Indeed, topical application of compounds with antioxidant properties such as curcumin-loaded poly (lactic acid) nanofibers^16^, chitosan-loaded eugenol^17^, and citrate-based hydrogels^18^ have demonstrated benefits in wound healing. However, compound biocompatibility and efficient delivery continue to pose significant challenges to their therapeutic use.

This clear clinical need led to our hypothesis that nature’s own potent antioxidant and proficient radical scavenger, melanin, could be mimicked synthetically and applied topically following skin injury, where it would be effective in healing when applied after chemical or UV-light exposure at the surface of the skin. This is based on the fact that melanin’s role as a pigment in the hair and skin^19,20^, is intrinsically linked to its photoprotective materials properties^20–22^. In brief, upon UV exposure, melanin expression in the skin is upregulated due to its ability to scavenge the free radicals generated by the impinging radiolytic light. Similarly, recent studies have reported increases in melanin-based skin pigmentation in response to air pollutants^23^.

To test this hypothesis, we synthesized two types of nanoscale Synthetic Melanin Particle (SMP) with Low and High Surface Areas (SMP_Lo_ and SMP_Hi_, respectively)^24^. As a result of their higher surface area, SMP_Hi_ were expected to exhibit a superior radical scavenging capability that would be reflected in wound healing efficacy compared to SMP_Lo_. The two types of SMPs were tested by topical application following chemical (nitrogen mustard, NM) and ultraviolet (UV)-induced skin wounds in vivo using mouse models^25^. In addition, SMPs were tested ex vivo using a human skin explant model for chemical-induced skin wounding. The data show that SMP treatment mitigates inflammation and accelerates wound healing by decreasing *Mmp9* expression^26^, rescuing skin superoxide dismutase (SOD) activity^27^, and suppressing chemical injury-induced MAPK signaling by inhibiting ERK1/2 phosphorylation. In a critical finding, SMPs were found to modulate the immune response by increasing the number of reparative and anti-inflammatory cells in the wound area and regulating the systemic immune response. Furthermore, in human skin explants SMP_Lo_ were shown to efficiently counteract the harmful effects of NM insult. The high-surface-area particles allowed for more exposure of the wound site to the melanin material and conferred stronger wound-healing benefits observable visually and pathologically in mice, and at the molecular level compared to the low-surface-area particles with both exhibiting efficacy over vehicle controls in a model-dependent fashion. Overall, the results demonstrate that biomimetic SMPs are an effective topical intervention for the acceleration of acute chemical- and UV-wound healing.

## Results

### SMP syntheses and characterization

**Fig. 1:**
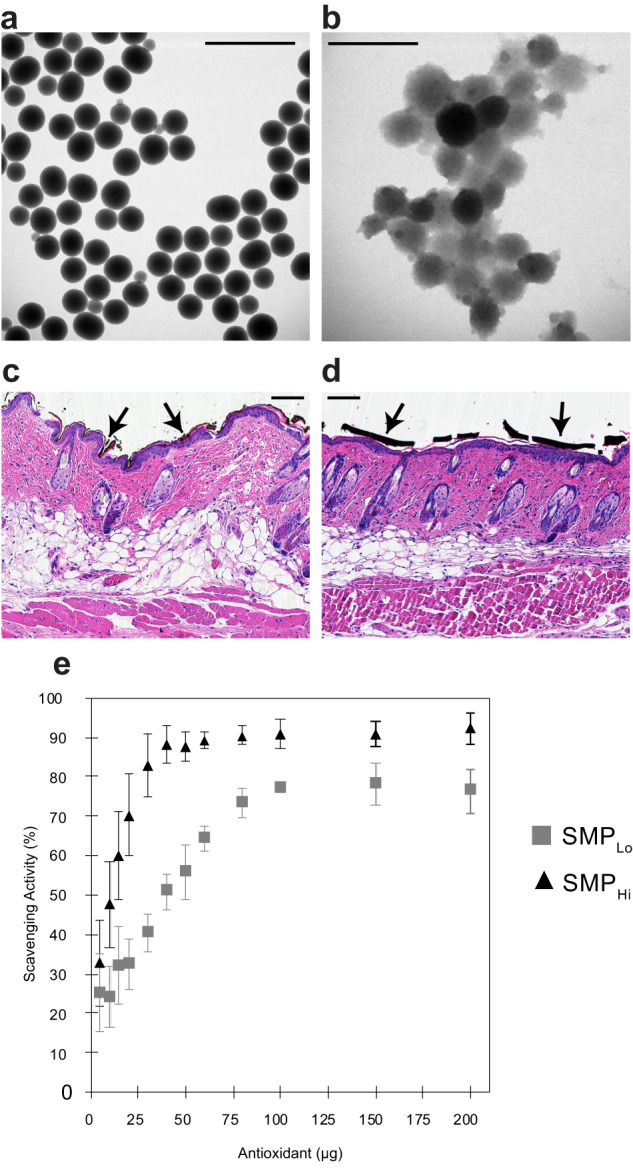
Characterization of high and low surface area synthetic melanin particles. **a**, **b** TEM micrographs of SMP_Lo_ and SMP_Hi_, respectively. Scale bars = 1 µm. **c**, **d** H&E stained optical micrographs of mouse skin sections with SMP_Lo_ and SMP_Hi_, respectively, shown residing on the surface of the skin as marked by black arrows. Scale bars = 100 nm. **e** 2.2-diphenyl-1-picrylhydrazyl (DPPH) radical scavenging activity of SMP_Hi_ (triangle) and SMP_Lo_ (square). Data are mean ± s.d.

Melanin mimetics were synthesized through the oxidative polymerization of dopamine, based on previously reported methods (Fig. 1)^24^. The surface area and morphology of the particles were characterized using N_2_ sorption, dynamic light scattering, and ultraviolet-visible (UV-Vis) spectroscopy (Supplementary Fig. 1). The High-surface-area Synthetic Melanin Particles (SMP_Hi_) had a BET area of 190 m^2^/g with approximately 14 and 30 Å pores while the Low-surface-area Synthetic Melanin Particles (SMP_Lo_) were non-porous with a BET area of 20 m^2^/g (Supplementary Fig. 1a, b)^24^. We note that the surface area of SMP_Lo_ is similar to that of natural melanin which has been measured to have a BET area of 6 m^2^/g as previously reported for melanin extracts from *Sepia officinalis*—squid ink^24^. By dynamic light scattering (DLS), SMP_Hi_ had a hydrodynamic diameter of 220 ± 50 nm. SMP_Lo_ had a hydrodynamic diameter of 320 ± 10 nm (Supplementary Fig. 1c). These scattering data are consistent with that observed by bright field TEM (Fig. 1a, b) Additionally, SMP_Lo_ and SMP_Hi_ exhibited similar absorption spectra by UV–Vis spectroscopy (Supplementary Fig. 1d) with both particles exhibiting an absorption maximum of approximately 200 nm with a shoulder at 300 nm and a broad absorption tail. Other than porosity, the two particles had similar characteristics. Neither SMP_Hi_ nor SMP_Lo_ penetrated the stratum corneum when applied topically to skin (Fig. 1c, d).

The scavenging activity of SMP_Hi_ and SMP_Lo_ was assessed via the 2.2-diphenyl-1-picrylhydrazyl (DPPH) assay (Fig. 1e). SMP_Hi_ achieved higher scavenging activity at lower particle concentrations than SMP_Lo_. The scavenging activity of both particles plateaued at approximately 100 μg, with SMP_Hi_ achieving a maximum at 90% scavenging activity and SMP_Lo_ reaching 80%. Scavenging activity was consistent across different batches of particles and was maintained but dampened over multiple use cycles (Supplementary Fig. 2). The scavenging activities of SMPs, especially SMP_Hi_, are similar or superior to radical scavenging activities of other antioxidants as determined via the DPPH assay, such as butylated hydroxytoluene^28^, luteolin^29^, eugenol^30^, and ascorbic acid^31^.

### SMP intervention improves skin healing after nitrogen mustard-induced injury

**Fig. 2:**
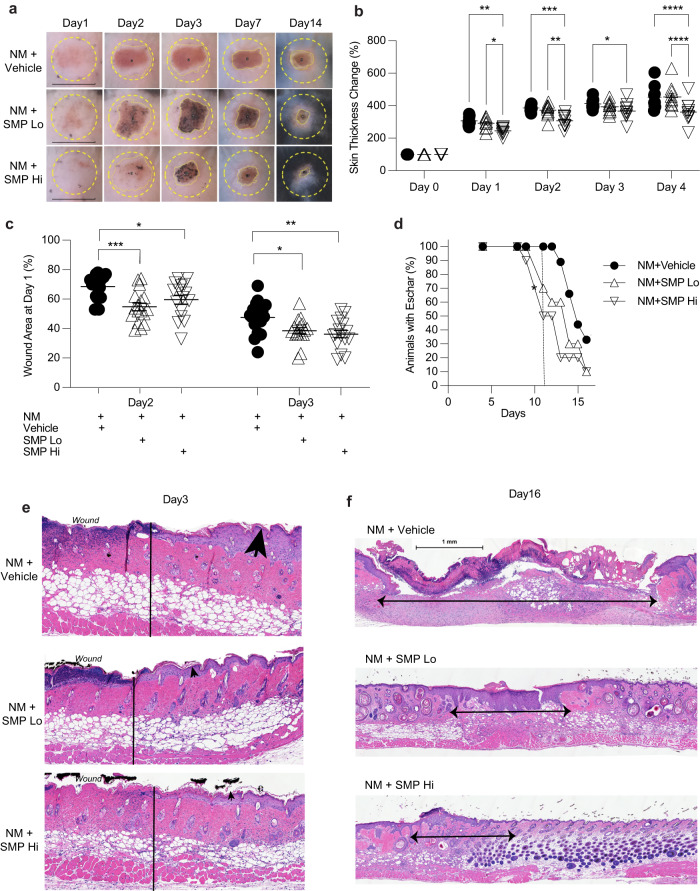
SMP intervention improves skin healing after nitrogen-mustard (NM) injury. **a** Representative images of the wounds on days 1 through 14. The dotted yellow line shows the area of NM application. Scale bars on day 1 images = 12 mm. **b** Bifold skin thickness measurements. Two-way ANOVA was used for multiple comparisons. *n* = 9–10 mice per group. **c** Wound area reduction. One-way ANOVA. *n* = 16–17 mice per group. **d** Time to eschar detachment. Gehan-Breslow-Wilcoxon analysis shows significance between Vehicle and SMP_Hi_ (asterisk). *p* = 0.03. *n* = 9*-*10 mice per group. **e** Representative images of skin samples collected at day 3 post-injury. The vertical line indicates the wound border. Arrowheads point to epidermal skin thickness. **f** Representative images of skin samples collected at day 16 post-injury. Arrows indicate the size of the wound. Hematoxylin and eosin staining, scale bar = 1 mm. *p < 0.05, **p < 0.01, ***p < 0.001 ****p < 0.0001. Data are mean ± s.e.m.

To test the hypothesis that intervention with topical SMP would improve wound healing, we utilized an NM-induced chemical injury mouse model (Fig. 2). NM is an alkylating agent that induces cell apoptosis via the formation of DNA interstrand crosslinks^32^. The clinical application of NM as a topical chemotherapeutic for the treatment of skin lymphoma is known to cause severe skin reactions including irritant dermatitis and blister formation^33^. In this experimental model, SMPs were applied topically to the NM-induced wound site 2 h after NM-induced injury to ensure that no residual NM was present on the skin surface at the time of SMP application. SMP application was then repeated at 24- and 48-h post-injury. The mice were monitored for up to 16 days.

Intervention with either type of SMP improved wound healing (Fig. 2a), evidenced by visible reduction in skin inflammation (erythema) in the early phase of tissue injury (days 1–2). SMP_Hi_-treated animals had decreased tissue swelling reaction as measured by edema (bi-fold skin thickness) (Fig. 2b). SMP-treated skin also healed faster than control skin as evidenced by faster rates of reduction in the radial wound area (Fig. 2c) and wound depth, as measured by time to eschar detachment (Fig. 2d). SMP_Hi_-treated animals healed more rapidly than both vehicle and SMP_Lo_ groups, with 50% of SMP_Hi_ mice demonstrating eschar detachment at 11 days post-injury compared to 30 and 0% in the SMP_Lo_ and vehicle groups, respectively. Statistical analysis showed a significant difference between SMP_Hi_ and vehicle treated groups (Fig. 2d). Skin sections were examined histologically at days 3 and 16 post-injury (Fig. 2e, f). The findings confirm decreased edema at day 3 as evidenced by epidermal thickness (Fig. 2e) as well as epidermal and dermal recovery at day 16. Here, we note the size of the healing wounds and the formation of full-thickness epidermis (Fig. 2f) in SMP-treated animals compared to vehicle control. To demonstrate the generality of the observed biological effect of SMPs, parallel experiments were conducted using a UV-radiation-induced injury model. Similarly, we observed that SMP intervention resulted in reduced skin edema and faster wound healing rates (Supplementary Fig. 3).

### SMPs downregulate inflammatory and apoptosis pathways and enhance angiogenesis

**Fig. 3:**
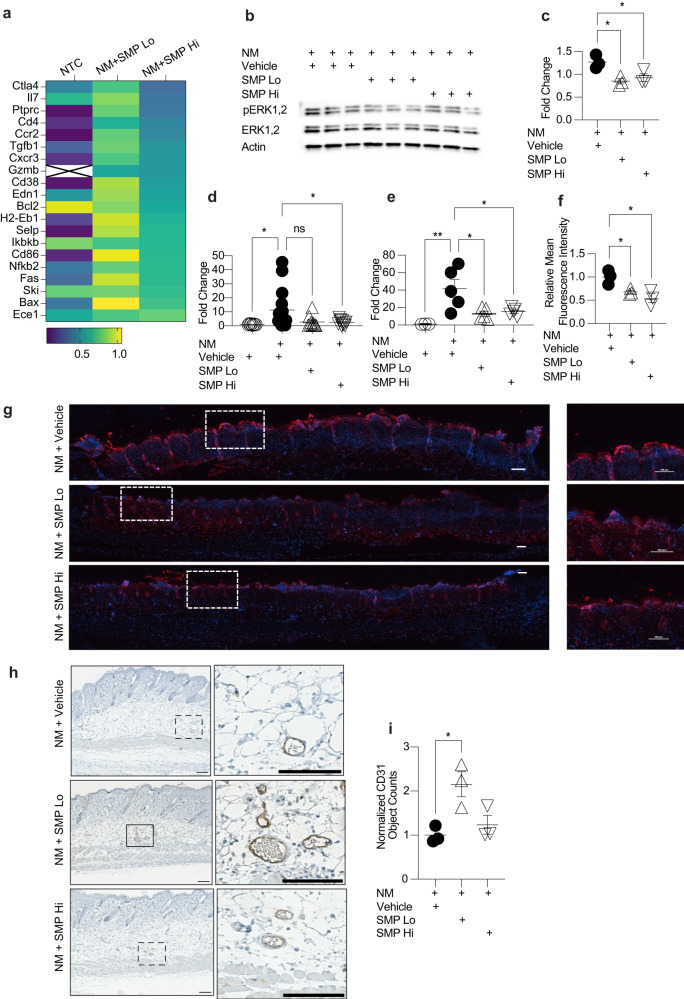
SMP intervention inhibits pro-inflammatory and pro-apoptotic pathways after nitrogen mustard (NM) injury. **a** TaqMan mouse immune qPCR array results relative to NM+Vehicle. Genes significantly downregulated in the NM+SMPHi treated group are shown as a heat map (one sample *t* test, all *p* ≤ 0.05). *Gzmb* values in the non-treated group were below the limit of detection. **b**, **c** Western blotting analysis of ERK1/2 phosphorylation 24 h after injury. **b** Western blotting image, (**c**) Densitometric quantification of data shown in (**b**). *n* = 3, *t* test, *p < 0.05. **d**, **e** Expression of *Mmp9* after 48 (**d**, *n* = 8–15 mice per group) and 72 (**e**, *n* = 3–5 mice per group) hours. One-way ANOVA, *p < 0.05, **p < 0.01. **f**, **g** SMP treatment downregulates pro-apoptotic signaling after NM-induced injury. **f** Mean fluorescence intensity of TUNEL staining. *n* = 3 mice per group, *t* test, *p < 0.05. **g** Representative images of TUNEL staining. Scale bar = 100 µm. **h**, **i** SMP treatment upregulates angiogenesis. **h** Representative images of CD31 staining. Arrows indicate the stained vasculature. **i** Normalized counts of CD31 staining compared to NM+Vehicle group. *n* = 3 mice per group, *t* test, *p < 0.05. Scale bar = 1 mm. Data are mean ± s.e.m.

To identify the pathways involved in SMP-induced wound-healing, we utilized TaqMan mouse immune arrays (Fig. 3). NM injury without SMP application led to upregulation of inflammatory genes while intervention with SMPs suppressed this upregulation, SMP_Hi_ treatment resulted in a more pronounced response than SMP_Lo_. Specifically, following NM-induced injury, treatment with SMP_Hi_ significantly downregulated the expression of 20 genes compared to the vehicle-treated animals (Fig. 3a, Supplementary Fig. 4). Analysis of the gene expression data using the PANTHER Classification System^34^ identified apoptosis signaling as the major pathway affected by SMP intervention; the genes determined by the classification algorithm were *Fas*, *Bax*, *Gzmb*, *Ikbkb*, *Nfkb2* and *Bcl2*. The other two main affected pathways were inflammation mediated by chemokine and cytokine signaling (*Cxcr3*, *Ccr2*, *Ikbkb*, *Nfkb2*) and T cell activation (*Cd86*, *Ptrpc*, *Ikbkb*, *Nfkb2*) (Fig. 3a). In the SMP_Lo_-treatment group, the general trend was also toward downregulation of inflammation-related genes (Supplementary Fig. 4). However, because of the high variability between individual arrays, only *Bcl2* demonstrated a statistically significant downregulation.

NM is reported to induce pro-inflammatory signaling through the mitogen-activated protein kinase (MAPK) pathway^35^. Accordingly, we performed western blotting analysis to determine the effect of SMP treatment on extracellular signal-regulated kinase 1/2 (ERK1/2) signaling. We found that treatment with either type of SMP significantly inhibited phosphorylation of ERK 1/2 at 24 h post-injury (Fig. 3b, c and Supplementary Fig. 9a–c).

Since MAPK-pathway signaling regulates inflammatory mediators at the translational and transcriptional levels, we sought to determine if SMP treatment led to expression changes in known NM injury-associated proinflammatory mediators. We found that the expression of *Mmp9* was significantly reduced by SMP_Hi_ at both 48 and 72 h after injury and by SMP_Lo_ at 72 h (Fig. 3d, e).

To confirm that SMP intervention inhibits apoptosis, we performed terminal deoxynucleotidyl transferase dUTP nick end labeling (TUNEL) staining of skin samples collected from each experimental group. As expected, NM injury alone resulted in strong TUNEL-positive staining (Fig. 3f, g). TUNEL staining was significantly reduced in mice treated with either type of SMP compared to the vehicle-only group by mean fluorescence intensity (*p* < 0.05 for both), indicating decreased apoptosis.

Since oxidative stress and ROS are known to influence the angiogenic response, we evaluated the effect of SMP treatment on the expression of CD31, a well characterized endothelial marker. Sections of the mouse skin from various experimental groups were immunoassayed and images quantified (Fig. 3h, i). Obtained data demonstrate that treatment of the injured skin with SMP_Lo_ resulted in a significant increase in angiogenesis.

### SMPs upregulate anti-inflammatory cells in the skin and decrease splenic immune cell populations

**Fig. 4:**
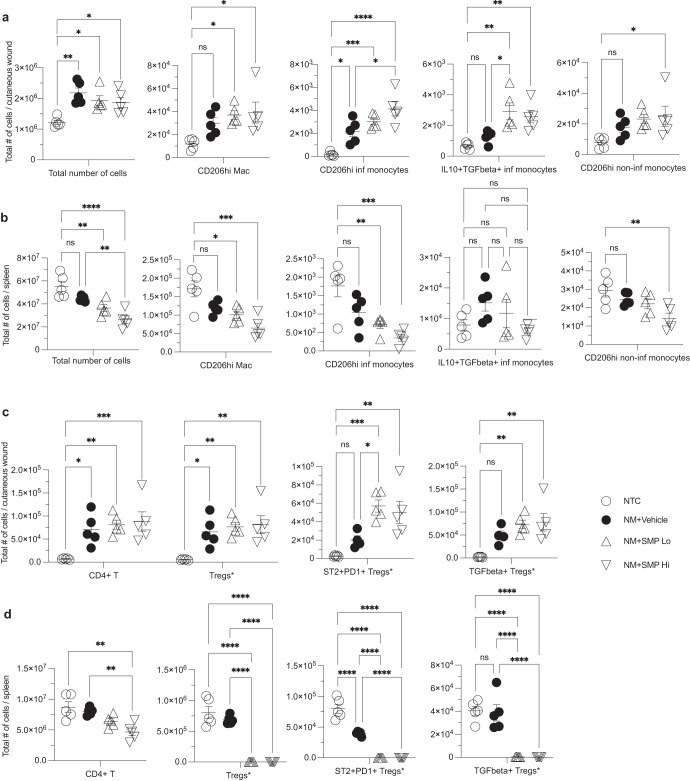
SMP particles significantly increase the infiltration of anti-inflammatory cells in cutaneous wounds. Panels (**a**) and (**b**) show APC cell population in skin and spleen, respectively. Panels (**c**) and (**d**) show lymphoid cells in skin and spleen, respectively. *Tregs are CD4 + CD25+FoxP3+ Tregs. One-way ANOVA was used for statistical analysis. *N* = 4–5. *p < 0.05, **p < 0.01, ***p < 0.001, ****p < 0.0001. Data are mean ± s.e.m.

Next, we sought to examine the acute immune response provoked by NM-injury which affects the trajectory of wound healing. To investigate the effect of SMP treatment on immune activation and infiltration we performed flow cytometric analysis of wounded skin on day 3 post-injury (Fig. 4a, c; Supplementary Figs. 5, 6). As expected, the total numbers of CD45+ immune cells in the wound area were increased in all experimental groups compared to uninjured mice (Fig. 4a).

In SMP-treated animals, the total number of immune cells trended slightly lower than in the NM-injured group, although the difference was found to not be statistically significant. The levels of antigen-presenting cells, including inflammatory monocytes, non-inflammatory monocytes, macrophages, pDCs and neutrophils were similar across all experimental groups (Supplementary Fig. 7). However, a more detailed analysis revealed that SMP treatment caused an increase in the proportions of CD206hi and IL10+TGFb+ monocytes and CD206hi macrophages (Fig. 4a). The analysis of infiltrating lymphoid cells in the skin showed that while there was no difference in the total numbers of CD4+ T cells and CD4 + CD25+FoxP3+ Tregs, SMP_Lo_ intervention led to a significant increase of ST2 + PD1+ Tregs compared to the NM-injured group. This trend was also apparent in TGFb expressing Tregs (Fig. 4c).

Given the important role of the spleen in regulating the response during acute inflammation, being a reservoir of immune cells, flow cytometric analysis of splenic tissue was concurrently performed in treated animals (Fig. 4b, d). Here, we observed a significant decrease of total immune cells in the SMP-treated groups, particularly pronounced in SMP_Hi_ group. Furthermore, the difference between non-treated and NM-injured animals was much smaller and not statistically significant (Fig. 4b). This trend was confirmed in inflammatory and non-inflammatory monocytes, macrophages and neutrophils (Supplementary Fig. 7).

To a remarkable extent, the dynamics of cell populations in the skin appears to be the mirror opposite of the spleen. In the skin we observed an increase in the CD206hi population of inflammatory monocytes, non-inflammatory monocytes, and macrophages, while in the SMP-treated mice (Fig. 4a), there was a concomitant steep decrease in the corresponding populations in the spleen (Fig. 4b). Similarly, in the spleen there was a significant reduction of PD1+, ST2 + PD1+, and TGFb+ CD4+ T cells in SMP-treated mice (Fig. 4d). Excitingly, these findings indicate that the topical application of SMPs alters the skin and the chemo-attraction of immune cells from systemic responses following skin injury.

### SMP intervention increases superoxide dismutase activity after NM-induced injury

**Fig. 5:**
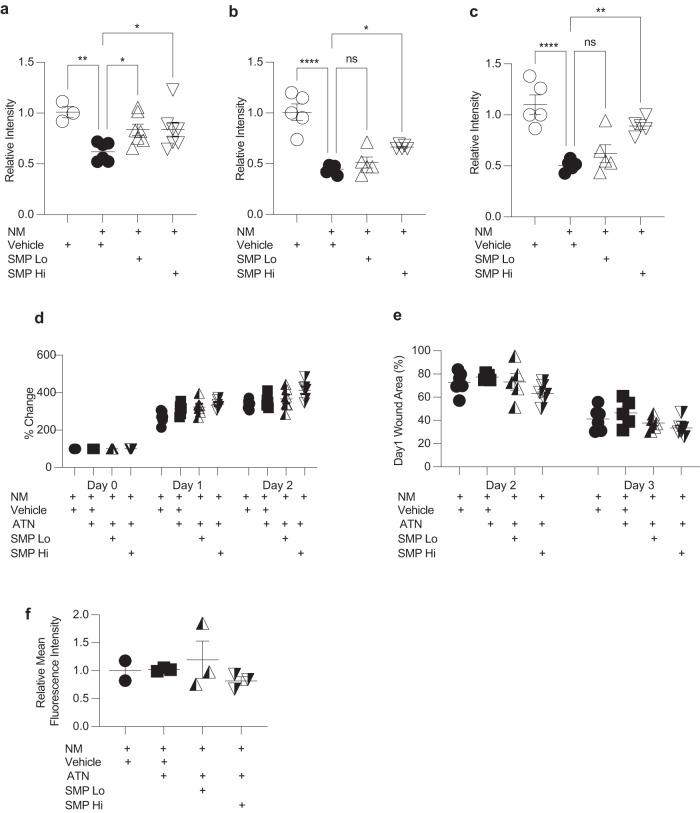
Role of SOD in SMP-mediated wound healing improvement after NM injury. **a**–**c** SMP intervention partially rescues SOD activity. **a** 24 h, *n* = 3–7 mice per group. **b** 48 h, *n* = 5 mice per group. c) 72 h, *n* = 5 mice per group. One-way ANOVA, *p < 0.05; **p < 0.01; ***p < 0.001. **d**–**f** Inhibition of Cu/Zn SOD abrogates the effect of SMP on skin healing after NM injury. **d** Bi-fold skin thickness measurements, *n* = 5-6 mice per group. **e** Wound area reduction, *n* = 5-6 mice per group. **f** Mean fluorescence intensity of TUNEL staining *n* = 3 mice per group. Data are mean ± s.e.m.

Skin injury leads to the excessive release of ROS. Antioxidant enzymes play a major protective role against the deleterious effects of ROS, and superoxide dismutase (SOD) is among the most important of these enzymes^36^. Therefore, we evaluated how SMP treatment affected SOD activity in skin samples collected at 24-, 48-, and 72-h post-NM-injury (Fig. 5). We found that NM-induced injury resulted in significant suppression of SOD activity compared to uninjured skin at all evaluated time points (Fig. 5a–c), while SMP intervention provided a statistically significant rescue of SOD activity. The effect of SMP_Hi_ treatment on SOD activity was more pronounced than that of SMP_Lo_.

We further investigated the activity of catalase and thioredoxin reductase, two other antioxidant enzymes important in skin, in response to SMP treatment. We did not find statistically significant differences in the activity of these enzymes between the experimental groups (Supplementary Fig. 8a, b).

### Inhibition of Cu/Zn SOD partially reverses SMP effects on skin wound healing

To determine whether the pro-healing effects of SMP were dependent on preserving SOD activity, we performed NM-injury experiments in the presence of the SOD inhibitor ATN-224, a small molecule copper-chelator and well-known inhibitor of SOD1. The application of ATN-224 largely abrogated the positive effects of SMP on wound healing. The degree of skin edema was similar across all treatment groups (Fig. 5d), as were the rates of wound healing (Fig. 5e). Although SMP_Hi_ appeared to slightly increase the rate of wound healing, there were no statistically significant differences between the groups at days 2 and 3 post-injury. Additionally, after ATN-224 administration, SMP failed to inhibit apoptosis as demonstrated by TUNEL staining (Fig. 5f). Thus, the inhibition of Cu/Zn SOD partially reversed the beneficial effects of SMP on skin wound healing.

### SMP_Lo_ protects human skin explants from NM-induced injury

With data in hand from mouse models, we next performed a proof-of-concept evaluation of the effect of SMP intervention on NM-injured human skin using ex vivo skin explants obtained from healthy donors following abdominoplasty surgery (n = 10, see Supplementary Table 1). This model provides valuable information concerning the keratinocyte population and pathophysiological behavior of the epidermal/dermal layers in a static manner. Static in the sense that, being an ex vivo model, explants do not have active circulation nor continuous influx of immune cells.

To assess the extent of NM-induced injury and effects of SMP treatment on the healthy human skin explants, blinded pathophysiological evaluation of hematoxylin- and eosin-stained slides was performed by a clinical dermatopathologist. NM-induced injury is characterized by rapid DNA damage followed by cell death and eventual blister formation. Therefore, the slides were evaluated by the following three histological criteria: (1) presence and degree of ballooning degeneration, spongiosis and epidermal pallor; (2) presence and number of dyskeratotic and apoptotic cells; and (3) presence and degree of subepidermal split. Criteria were scored on a scale of 0 to 2, with 0 representing an absence, and 2 representing a pronounced appearance of the evaluated characteristics. These criteria reflect the sequential order of NM-induced injury in the skin with skin blister formation representing the cumulative damage.

**Fig. 6:**
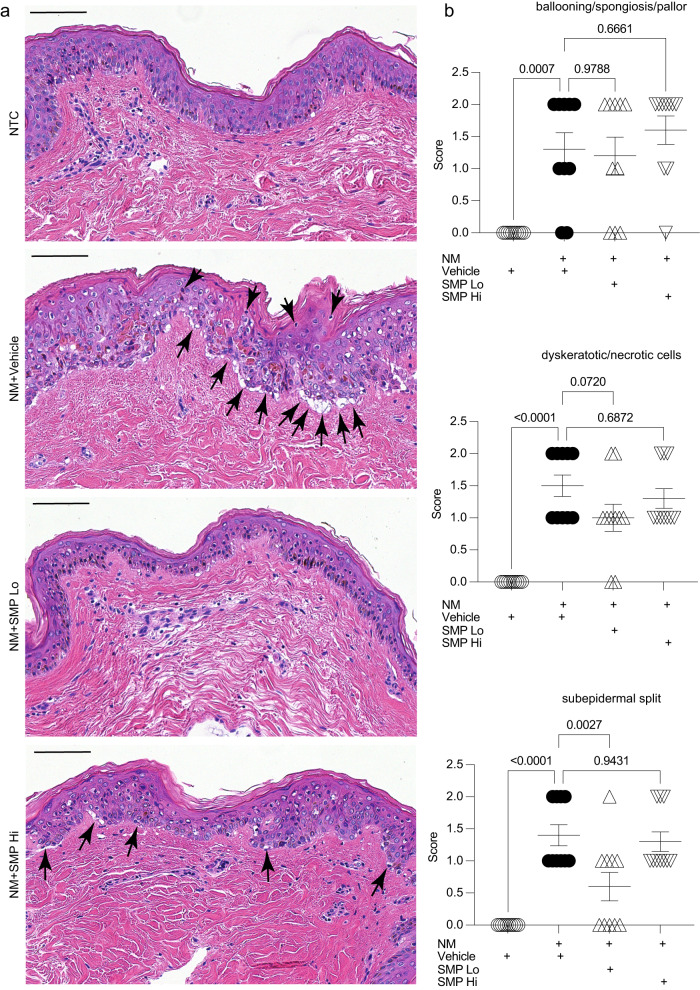
Nitrogen-mustard (NM) induced skin injury in human explants. **a** Representative images of the wounds. Hematoxylin and Eosin staining. Scale bar=100 µm. **b** Dermatopathological scoring of NM injured skin. One-way ANOVA. *n* = 10 subjects. *P* values are shown on the graphs. Arrows indicate tissue damage by NM injury. Data are mean ± s.e.m.

As expected, treatment of healthy skin explants with NM resulted in a significant increase of all three scored criteria (Fig. 6). While there was no significant effect of SMPs on ballooning degeneration, spongiosis, and epidermal pallor, the explants treated with SMP_Lo_ showed noticeable decreases in the number of dyskeratotic/apoptotic cells, with 20% of subjects demonstrating no dyskeratotic/apoptotic cells, as opposed to 100% of subjects having at least some presence of these cells in the vehicle group. Even more impressive results were obtained after the evaluation of the third criterion. In SMP_Lo_ treated explants, subepidermal split was completely prevented in 50% of explants, while it was present in all tissues in the vehicle group. SMP_Hi_ particles had a mild but non-statistical effect on sub-epidermal split mitigation.

## Discussion

Topical application of Synthetic Melanin Particles (SMPs) improved skin healing after chemical and UV-induced injury. Melanin is a natural polymeric biomaterial that plays a role in skin protection against radiation^20^ and toxin insult^23^. Exposure to such harmful conditions often leads to an increase in melanin production in the skin as a natural response that capitalizes on melanin’s protective role. Due to its electron-rich functional groups, melanin has complex redox properties, allowing it to scavenge radical oxygen species and adsorb other harmful molecules that are generated in the skin during exposure^37,38^. Therefore, we reasoned that a biocompatible radical scavenging melanin-mimetic material presents an appealing topical intervention to promote tissue repair after chemical or UV injury.

SMPs were synthesized to mimic native melanin in the skin. Previous work with melanin nanoparticles similar to that of the Low Surface Area Synthetic Melanin Particles (SMP_Lo_) demonstrated no toxicity or significant changes in cell viability in 2D cell cultures of human keratinocytes and an increase in protection against cell death from UV-induced damage^22^. High Surface Area Synthetic Melanin Particles (SMP_Hi_)^24^, with increased exposure of the material to the surroundings, and therefore greater scavenging capacity, were synthesized to compare to SMP_Lo_ and further elucidate melanin’s role in wound healing especially surrounding reactive oxygen species (ROS).

During homeostasis, basal levels of ROS are tightly regulated and are involved in maintaining normal cellular function^39^. However, tissue injury from UV-irradiation or toxic chemical insult, such as in the case of nitrogen mustard (NM), results in the release of excessive amounts of ROS and oxidative stress. ROS produced during oxidative stress directly oxidizes membrane lipids, enzymes, structural proteins and nucleic acids, leading to cell death or improper cellular function^40^.

One of the main antioxidant enzymes in mammals is SOD, which converts superoxide radicals into hydrogen peroxide. The SOD family has three isoforms: Cu/Zn SOD1, which is located in the cytoplasm, nuclei, and intermembrane space of mitochondria; Mn/Zn SOD2, which resides in the mitochondrial matrix; and Cu/Zn SOD3, which is secreted extracellularly^36,41^. Mice lacking *Sod2* are not viable, dying within 21 days of birth. *Sod1* knock-out mice are largely normal but display delayed wound healing. In addition, *Sod1* knockdown induces senescence in human fibroblasts^42^ and is necessary for maintaining mouse embryonic fibroblasts in cell culture^43^. Mice with knocked out *Sod3* appear normal but have reduced survival time under high oxygen tension^36^. Thus, the rapid removal of highly reactive superoxide radicals by SOD is necessary for maintaining redox homeostasis and proper cellular survival and function. Additionally, the hydrogen peroxide molecules produced by SOD play a bactericidal/bacteriostatic role and act as secondary messengers. As a small uncharged molecule, hydrogen peroxide easily diffuses through membranes and tissues, which allows it to act as a chemoattractant for inflammatory cells and promote proliferation of keratinocytes, fibroblasts, and vascular endothelial cells^12,44,45^.

Given the role of SOD in wound healing, it is likely that the rescue of SOD activity by SMP underlies the beneficial effects of melanin intervention following NM-injury. The protection of SOD activity by SMPs may contribute to wound healing in three ways: by directly removing superoxide radicals, by reducing the bacterial burden in the wound area, and by indirectly regulating multiple signaling pathways via hydrogen peroxide production. In terms of our mechanistic understanding, it is known that SOD itself is susceptible to inactivation by ROS^46^. Therefore, we postulated that by scavenging the excess ROS produced during skin damage, SMPs would protect SOD from ROS-induced inactivation. Indeed, our data show that inhibition of Cu/Zn SOD with Cu chelator ATN-224 resulted in the dampening of the positive effects of SMP intervention, confirming the critical role of Cu/Zn SOD. Since a lack of SOD1 is known to delay wound healing, we dosed the SOD inhibitor intentionally to produce a relatively mild effect on wound healing as not to completely mask the effect of SMP on the healing process.

Stimulation of MAPK pathways leads to the activation of multiple cellular events, including inflammatory responses, apoptosis, migration, and proliferation. The exposure of mouse skin to vesicants such as sulfur mustard, NM, and 2-chloroethyl ethyl sulfide (CEES) results in MAPK phosphorylation, suggesting that MAPKs play a role in vesicant-induced inflammation. Kumar et al.^35^ found that skin levels of pro-inflammatory molecules, such as TNFα, iNOS, Mmp9, were significantly increased following NM injury in the mouse. Phosphorylation of Erk1/2, p38 and Jnk1/2 are also found to be increased. ROS has also been implicated in MAPK activation^47^. Our data fits well in this context, demonstrating that decreasing the ROS burden with SMPs decreases pro-inflammatory signaling in the injured mouse skin, decreases Erk1/2 phosphorylation, and suppresses apoptosis.

Melanin nanoparticles have been reported to decrease ROS and inflammatory processes after being actively engulfed by macrophages^48^. However, in our study, SMPs were applied topically and were not found to penetrate the stratum corneum. The beneficial effects of SMP intervention, such as decreased skin edema and increased SOD activity, were found to occur within 24 h of application before NM-induced skin injury was clinically evident. These observations argue that the efficacy of SMPs was not dependent on cellular uptake of the melanin particles by macrophages or another cell type. Although not observed, if a subset of particles penetrated the skin as a consequence of NM-inflicted damage, the positive effect of the initial SMP application cannot be explained by the direct cellular uptake of the melanin particles.

H_2_O_2_ is one of the main secondary messengers during wound healing response. It is reported to regulate angiogenesis, stimulating proliferation of vascular endothelial cells^39,44^. Indeed, SMP_Lo_ particles significantly increased angiogenesis after NM injury. The failure of SMP_Hi_ particles to influence the angiogenic response is surprising and merits further investigation.

The roles of the CD206 mannose receptor positive anti-inflammatory M2 macrophages and Tregs in the resolution of inflammation are well accepted^3,4,49–51^. CD206 M2 macrophages dampen inflammation in part by converting pro-inflammatory M1 macrophages towards an M2 phenotype through cell-cell contact and soluble factors^52^. We found that topical application of SMPs not only increases CD206 M2 macrophages in wounded skin, but also their communication with systemic inflammation as observed in the splenic populations of the NM-injured animals. The spleen plays a crucial role during an immune response, being an organ for immune cell proliferation and a reservoir of immune cells. Intervention with SMPs may have a direct effect on stabilizing the skin thus altering its chemokine expression and attraction of immune cells from the spleen and circulation. Furthermore, the data suggests skin in situ activity as splenic inflammatory monocytes did not express anti-inflammatory cytokines IL-10/TGFb. They are only observed in the skin consistent with maturing CD206 monocytes acquiring the pro-resolution characteristics as they enter a target microenvironment^53^. Fittingly, it was reported that in NM-exposed rats, the splenectomy was responsible for increasing M1 macrophages and decreasing subsets of M2 macrophages, resulting in exacerbated tissue injury^54^. The relationship between CD206 M2 macrophages and regulatory T cells is complex. In vitro, CD206 M2 macrophages can generate Tregs through cell-cell contact while in vivo, therapeutic infusion of CD206 M2 macrophages increases splenic regulatory T cells^52^. The latter was observed in a nephritis injury model at 21 days (3 weeks) suggesting de-novo Treg generation. By contrast, our acute injury model shows an increase in skin with concomitant decrease in splenic Tregs at day 3 suggesting mobilization and re-distribution rather than de novo Treg generation.

The alteration of local and systemic immune responses thus plays a key role in the protective function of SMPs. In human explant experiments, SMP_Lo_ particles demonstrated impressive mitigation of skin tissue damage and prevention of blisters after NM-injury. Topical application of SMP_Lo_ prevented both cellular damage and the cumulative effect of epidermal-dermal separation. The absence of any effect of SMP_Hi_ particles on skin explants may reflect the importance of immunological response in SMP_Hi_ signaling. Indeed, since the ex vivo explants do not possess a functional circulation, the effect of SMP_Hi_ may be diminished. These differences between particle type may be potentially attributed to the morphology of the particles and how they interact with the stratum corneum as a result; a finding that requires further investigation and optimization in subsequent testing. Furthermore, the skin explant model devoid of immune cells will be useful for investigations to assess chemokines and factors produced by the epithelial barrier critical for the recruitment of destructive or reparative immune cells.

We predicted that an increase in the surface area of the melanin particles would contribute to their antioxidant capacity and tissue healing ability. SMP_Hi_ has higher radical scavenging activity at lower concentrations than SMP_Lo_ and demonstrated superior performance in wound repair. Generally, synthetic melanin particles have several distinct features that make them an excellent candidate for therapeutic applications. Being a natural product biomimetic, they are inherently biocompatible and non-allergenic. In addition, their surface charge, size, and surface area may be modified, allowing for ease of delivery onto the site of injury^55,56^. The tunable surface area also creates numerous possibilities for the melanin particles, broadening their applications beyond wound-healing targets. Taken together, our data demonstrate that synthetic melanin nanoparticles significantly improve wound healing and have strong potential to be developed as therapeutic agents.

## Methods

### Materials

Tetraethyl orthosilicate (TEOS) and 25 wt% poly(acrylic acid) solution (PAA) were purchased from Acros Organics. Hexadecyltrimethylammonium bromide (CTAB) was purchased from Tokyo Chemical Industry (TCI). Dopamine hydrochloride was obtained from Alfa Aesar. Ammonium hydroxide was purchased from Fisher Scientific. Hydrofluoric acid (HF), ethanol, and Trizma®Base (tris) were obtained from Sigma Aldrich. All materials were used as received without further purification.

### Preparation of SMP_Lo_

SMP_Lo_ was synthesized through the oxidative polymerization of dopamine. Briefly, 900 mg of dopamine was dissolved in 300 mL of ultrapure water and 4.2 mL of 1 M NaOH was added to the solution at room temperature and stirred for 18 h. The solution was then centrifuged and washed with ultrapure water 5 times.

### Preparation of mesoporous silica template

Mesoporous silica nanoparticles (MS) used as a template were synthesized based on a previously reported literature method^57^. Briefly, 0.55 g CTAB and 3.00 g PAA were dissolved under 25 mL of ultrapure water and stirred vigorously until the solution was clear. 2.0 g ammonium hydroxide was added to the stirring solution. After 20 min, 2.08 g TEOS was added and stirred for an additional 15 min. Then the solution was placed in an oven for 48 h at 120 °C. The mixture was then centrifuged and dried before calcining the particles for 6 h at 550 °C to remove the remaining organic template.

### Preparation of SMP_Hi_

SMP_Hi_ was prepared based on a reported literature method^24^. 250 mg of MS was sonicated for 1 h in ultrapure water. To 225 mL of ultrapure water and 25 mL of ethanol (9:1 H_2_O:EtOH by volume), 250 mg MS and 225 mg dopamine was added and stirred for 1 h at room temperature. Tris (10 mM, pH 8.5) was then added to the reaction and stirred for an additional 4 h. After the allotted time, the reaction was centrifuged and washed with ultrapure water 5 times. The template was removed through hydrofluoric acid (10 wt%) etch overnight, and then centrifuged and washed with ultrapure water 5 times.

### DPPH assay for radical scavenging activity

DPPH radical scavenging activity of SMPs was determined according to a reported literature method^58^. 100 µL of SMP dispersed in water was added to a 1.8 mL solution of DPPH (0.2 mM in 95% ethanol). The total amount of particles was varied from 5 to 200 µg. The solutions were left in the dark for 20 min. Afterwards, the scavenging activity was monitored by taking the absorbance of the solutions at 516 nm. To determine the DPPH radical scavenging activity the following calculation (Eq. 1) was used.

1 $$I=\frac{1-\left({A}_{i}-{A}_{j}\right)}{{A}_{c}}\times 100 \%$$

*I* is the DPPH radical scavenging activity, *A*_*i*_ is the absorbance of the samples with DPPH, *A*_*j*_ is the absorbance of the samples without DPPH, and *A*_*c*_ is the absorbance of the DPPH without the PDA samples.

### Particle characterization

All SMPs were characterized by transmission electron microscopy (TEM, Hitachi HT-7700, 120 KV, or STEM, Hitachi HD-2300A, 200 KV). Dynamic light scattering (DLS, Malvern Instruments Ltd, Nano ZS), zeta-potential (Malvern Instruments Ltd, Nano ZS), and ultraviolet-visible spectroscopy (UV-Vis, Agilent Technologies Cary 100 UV-Vis) were used to investigate hydrodynamic diameters.

### Sample activation for surface area measurements

Using a Micromeritics Smart VacPrep, samples were activated thermally under vacuum at 100 °C for the mesoporous silica and 75 °C for the pre-etched SMP_Hi_. Samples were activated using a tousimis SAMDRI-PVT-3D Advanced Manual Critical Point Dryer. Prior to activation, samples were exchanged into ethanol overnight. Using the supercritical dryer, the sample was added to the sample chamber, cooled to 0–10 °C, and pressurized to 800 psi. The ethanol was exchanged with liquid CO_2_ over the course of 10 h, purging the system for f5 min every 2 h. After the fifth purge, the temperature was raised to 40 °C and the system was pressurized to 1200–1400 psi. The pressure was released slowly overnight at a rate of 0.5 cc/min. Samples were immediately transferred onto a Micromeritics Smart VacPrep and were placed under vacuum for 2 h at 25 °C prior to sorption measurements.

### Nitrogen isotherms

For SMP samples, N_2_ isotherms were collected on a Micromeritics ASAP 2420 instrument at 77 K. Pore-size distributions were obtained using density functional theory (DFT) calculations with a carbon slit geometry and a N_2_ DFT model.

### Animals

All animal studies were conducted in accordance with NIH guidelines for the care and use of laboratory animals and protocols were approved by the Institutional Animal Care and Use Committee of Northwestern University. Six to eight-week-old C57BL/6J female mice were purchased from Jackson Laboratories.

### Nitrogen mustard skin injury model

Mice were exposed to nitrogen mustard (NM) as described previously^59^. Briefly, the dorsal area of mice was shaved and chemically depilated 48 h before skin injury induction. Mice were anesthetized and placed on a heating pad under a chemical fume hood. 0.5% (w/v) of mechlorethamine hydrochloride (nitrogen mustard, Sigma, #122564) solution in 1.5% DMSO-PBS was prepared immediately before the application. A total of 40 µl of NM solution was applied on a circular (12 mm diameter) area in two consecutive applications. After the application mice were placed in a temporary housing space under a chemical fume hood for 2 h.

### UV radiation skin injury model

Mice were exposed to UV radiation as described previously^60^. Briefly, a 12 mm diameter circular area of back skin depilated of hair was exposed to UVB irradiation from six FS-40 fluorescent lamps filtered through Kodacel (Eastman Kodak Co., Rochester, NY). UVB emission was measured with an IL-443 phototherapy radiometer (International Light, Newburyport, MA) furnished with an IL SED 240 detector. Mice were exposed to a single UVB dose of 100 mJ/cm^2^ to induce skin inflammation.

### Synthetic melanin particles intervention

SMPs were diluted in milli-Q water at concentration of 50 µg/µl. A total amount of 1 mg of particles were applied to the injured skin area 2 h after the injury induction, and then 24 and 48 h later. Milli-Q water was used as a vehicle. During the treatment mice were anesthetized using isoflurane.

### Monitoring skin injury and measurement of wound healing

Mice were examined non-invasively after induction of skin injury. Monitoring (including photographic images, bi-fold skin thickness and body weight measurements) was performed daily, starting on the day of skin injury. Photographs of the injured area were taken using a fixed-position camera. The bi-fold skin thickness of the injured area was measured using digital calipers (Mitutoyo, PK0505CPX). The area of the inflammation/wound was measured using Image J and QuPath software.

### Ex vivo human skin explant cultures, NM treatment, and SMP intervention

De-identified and discarded surgically resected human skin tissue was collected at Northwestern Memorial Hospital from abdominoplasty surgeries by the Northwestern University Skin Biology and Diseases Resource-based Center. The skin tissue was subsequently obtained from the Northwestern University Skin Tissue Engineering and Morphology Core. All tissues were collected in compliance with the Northwestern University Internal Review Board (#STU00009443).

The explants were collected from the skin samples of patients of various ethnicities, with an age range 25–51 years, median age ± st. dev. 36.6 ± 6.4 years (supplementary Table 1). Full-thickness punches (*d* = 12 mm) were collected, rinsed in sterile PBS, and after removal of the subcutaneous layer of fat placed onto stainless steel mesh platforms in a 60 mm petri dish. The RPMI-1640 media (Corning, #10–040-CM) supplemented with 10% FBS and penicillin/streptomycin (200 U/ml and 200 µg/ml, respectively) was added to the dishes so that the top of the mesh platform with explants remained above the level of media. The explants were incubated at 37^o^C and 5% CO_2_ overnight. After the incubation, freshly prepared 0.5% (w/v) solution of nitrogen mustard in a Clinique Dramatically Different^TM^ Moisturizing Gel/PBS (2 volumes of Gel/1 volume of PBS) was applied on the explant surface in two consecutive applications (total volume 40 µl). SMPs were applied as described above. The explants were kept in the incubator at 37^o^C and 5% CO_2_ for up to 48 h. The media were changed daily.

The extent of NM-induced injury and the effects of SMP intervention on skin explants were assessed by a blinded clinical dermatopathologist. Three histological criteria were used: (1) presence and degree of ballooning degeneration, spongiosis and epidermal pallor; (2) presence and number of dyskeratotic and apoptotic cells; and (3) presence and degree of subepidermal split. Criteria were scored on a scale of 0 to 2, with 0 representing an absence, and 2 representing a pronounced appearance of the evaluated characteristics.

### SOD, catalase and thioredoxin reductase activity measurements

Superoxide dismutase activity was measured using commercial SOD assay kit (Cayman Chemical #706002) following the manufacturer’s protocol. Briefly, frozen tissue samples were homogenized using PowerLyzer 24 Homogenizer (Qiagen) in cold 20 mM HEPES buffer (pH 7.2, containing 1 mM EGTA, 210 mM mannitol and 70 mM sucrose) and centrifuged at 10,000 *g* for 15 min at 4 °C. Resulting supernatant was assayed for SOD activity using a tetrazolium salt for detection of superoxide radicals. Absorbance was measured using a Victor plate reader (Perkin Elmer, Inc.). Catalase and thioredoxin reductase activity was measured using commercially available kits (Cayman Chemical #707002 and #10007892, respectively) following the manufacturer’s protocols.

### ATN-224 treatment

Animals were treated with ATN-224 (Cayman chemical #23553) in sterile saline at the dose of 4.5 mg/kg/day by oral gavage. The treatment was performed on days 0, 1, and 2 immediately prior to the SMP application. Vehicle group received saline.

### Western Blotting

Mouse skin samples were mechanically disrupted in ice cold RIPA buffer (ThermoFisher, 89900). The protein homogenates were heated to 95 degrees C for 5 min in 6x Laemmli SDS sample buffer (ThermoFisher, J61337.AC) with 10% 2-Mercaptoethanol (Sigma-Aldrich, M6250) then electrophoresed using a 4–15% Mini-PROTEAN® TGX™ precast protein gel (Bio-Rad, #4561083) and transferred to a 0.45 µm pore size PVDF blotting membrane (Cytiva, GE10600023). Blots were incubated with primary antibody overnight at 4 °C. Primary antibodies used in this study were purchased from Cell Signaling (#4695, #4377, # 4970; 1:1000 dilution). Blots were then incubated for 1 h at room temperature with horseradish peroxidase-labeled secondary antibody (Cell Signaling, #7074, 1:1000 dilution). Proteins were visualized by chemiluminescence (GE Healthcare, RPN2232). All blots were processed in parallel and derived from the same experiment.

### qPCR

Skin injury and wound healing-related gene expression profiling were measured with real-time quantitative PCR. Tissue samples were homogenized using PowerLyzer 24 Homogenizer (Qiagen) and total RNA was extracted from skin tissue using RNeasy Fibrous Tissue Mini Kit (Qiagen, 74704) according to the manufacturer’s protocol. TaqMan Gene Expression assays were used for measuring the relative gene expression levels of *Mmp9* (Mm00442991_m1). The relative expression fold change was calculated using the ddCt method. QuantStudio 7 real-time PCR System (ThermoFisher) was used for running quantitative PCRs.

### TaqMan immune array

TaqMan Mouse Immune Array v2.1 (Applied Biosystems #4365297) was used to obtain the differential gene expression profile of immune response-related genes. Experiments were performed according to the manufacturer’s protocol. Since each array card has a capacity for four samples and we had four experimental groups of animals (i.e., no treatment, NM+vehicle, NM+SMP_Lo_ and SMP_Hi_), to increase the number of samples assayed we pooled cDNA samples of three mice in each experimental group per array card. The arrays were repeated three times, in total using cDNA pooled from nine different mice in each experimental group, except for “no treatment” group, where the same three cDNA samples were used repeatedly. This “no treatment” group data was used to correct for the batch effects of the Ct values obtained from three independent arrays. The ddCt values were calculated using NM+vehicle group data as a reference, and one-sample t-test analysis was used to calculate the p values.

### TUNEL staining

The skin samples from euthanized mice were taken and fixed in neutral buffered 10% formalin diluted in PBS (Caplugs, 308–1101-G8T). Samples were then embedded in paraffin, sectioned (8-μm thickness), and stained. The coverslip was mounted using antifade mounting medium with DAPI (Vectasheild H-1200–10). The TUNEL assay was performed using the CF®640 R Dye TUNEL assay apoptosis detection kits (Biotium, 30074) according to the manufacturer’s directions and visualized with an ECLIPSE Ti2 inverted microscope (Nikon Tokyo, Japan) using a DS-Qi2 camera (Nikon). TUNEL staining was quantified by drawing a region of interest around the epidermis ensuring removal of any sectioning artifacts such as tissue tears and folding from the analysis. The fluorescence intensity of the TUNEL staining was normalized by the fluorescence intensity of the DAPI staining within the region of interest.

### CD31 staining

The skin samples from euthanized mice were taken and fixed in neutral buffered 10% formalin diluted in PBS (Caplugs, 308–1101-G8T). Samples were then embedded in paraffin, sectioned (8-μm thickness), and stained for CD31 and using eosin. Staining was done by the Northwestern University Mouse Histology and Phenotyping Laboratory. CD31 & eosin stained slides were visualized with a TissueFAXS Histo inverted microscope (TissueGnostics GmbH Vienna, Austria). Staining was quantified by drawing a region of interest adjacent to the wound area for the purpose of eliminating nonspecific CD31 stained areas. The ImageJ DAB staining color deconvolution function was used to separate eosin and DAB staining. The number of objects positive for DAB staining were normalized by the number of objects positive for eosin staining.

### Flow cytometry

Mouse skin samples were digested and dissociated cells were stained and analyzed by flow cytometry as described elsewhere^59^. The numbers of each subpopulation in the skin and spleen were determined by multiplying the percentage of lineage marker–positive cells by the total number of mononuclear cells isolated from the corresponding tissue.

Single cells were incubated with Fc block (anti-mouse CD16/32, 0.25 μg; eBioscience, San Diego, CA), washed with FACS buffer (PBS with 2.5% fetal bovine serum and 0.1% NaN_3_), and were then stained for surface markers using the specified antibodies in the table below. Cells were then washed with PBS, and viability staining was performed using the LIVE/DEAD fixable dead cell stain kit (Invitrogen, Carlsbad, CA). Following viability staining, cells were washed with PBS and were either resuspended in FACS buffer for flow cytometric analysis or were subjected to intracellular staining to detect inducible CD206, or FoxP3. For intracellular staining, cells were fixed and permeabilized using the FoxP3 staining buffer kit (eBioscience) and then intracellularly stained. As controls, fluorescence minus one (FMO) was used to place the gates for analysis.

For flow cytometric analysis, cells were first gated according to forward and side scatter and then restricted to single cells and live cells. Tissue infiltrating myeloid cells were identified as CD45^+^CD11b^+^CD3^−^ and infiltrating lymphoid cells as CD45^+^CD11b^−^CD3^+^ for T cells and CD45^+^CD3^−^CD11b^−^B220^+^CD11c^−^ for B cells. On the infiltrating lymphoid population, cells were gated on CD3^+^CD4^+^CD8^−^ or CD3^+^CD8^+^CD4^−^ to evaluate the different T lymphocyte subpopulations. For infiltrating myeloid cells, Ly6G^+^ neutrophils were first gated and excluded from the infiltrating myeloid subpopulations. The Ly6G^−^ myeloid cells were divided into CD11c^+^ and CD11c^−^ monocytes/macrophages. Finally, the monocytes/macrophages were further divided into Ly6C^hi^ inflammatory monocytes and Ly6c^lo^ noninflammatory monocytes. Expression of CD206 was evaluated on the monocyte/macrophage subpopulation.

Mouse-specific antibodies used are listed in Table 1. The dilutions were made according to the antibody concentrations. For 2 mg/ml concentration, 1:100 dilution was used. For different concentrations, the dilution was adjusted accordingly. AbA 6-laser Fortessa flow cytometer (BD Biosciences) was used to enumerate cell populations and the data was analyzed using FlowJo software (TreeStar, Ashland, OR).

**Table 1:** Antibodies used for flow cytometry.

SPECIFICITY	SOURCE	CAT #
CD11b	BioLegend	101257
CD11c	BioLegend	561241
CD25	BioLegend	102008
CD3	BioLegend	560771
CD4	BioLegend	300532
CD45	BioLegend	561487
CD8	BioLegend	100730
IL-10	BioLegend	17-7101-81
Ly6C	BioLegend	128016
Ly6G	BioLegend	127627
PD-1	BioLegend	135220
ST2	BD Biosciences	745403
TGF-β	BioLegend	141414
B220	BD	557957
CD206	BioLegend	141721
FoxP3	BioLegend	45-5773-82

### Statistical analysis

GraphPad Prism V.8.3.0 software (San Diego, CA) was used to create visual graphics and to calculate the statistical significance. One-way ANOVA, chi-square and *t* test were used to calculate the *p*-values.

### Reporting summary

Further information on research design is available in the Nature Research Reporting Summary linked to this article.

### Supplementary Information

Supplemental Data

Reporting Summary

## Data Availability

The data that supports the findings of this study are available from the corresponding authors upon reasonable request.
