# Supplementary material for: Topical application of synthetic melanin promotes tissue repair
Source: NPJ Regen Med. 2023 Nov 2;8:61. doi: 10.1038/s41536-023-00331-1 (PMC10622536; doi:10.1038/s41536-023-00331-1)
Supplement: Supplementary file 1 — Supplemental Data [file 41536_2023_331_MOESM1_ESM.pdf]

Supplemental Data

Topical Application of Synthetic Melanin Promotes Tissue Repair

Dauren Biyashev[1,10], Zofia E. Siwicka[2,3,10], Ummiye V. Onay[1], Michael Demczuk[1], Dan Xu[7], Madison K. Ernst[1], Spencer T. Evans[1], Cuong V. Nguyen[1], Florencia A. Son[2,3], Navjit K. Paul[2,3], Naneki C. McCallum[2,3], Omar K. Farha[2,3,4], Stephen D. Miller[7], Nathan C. Gianneschi[2,3,5,6,8,9,*], Kurt Q. Lu[1,*]

[1]Department of Dermatology, Feinberg School of Medicine, Northwestern University, Chicago, IL.
[2]Department of Chemistry, Northwestern University, Evanston, IL.
[3]International Institute of Nanotechnology, Simpson-Querrey Institute, Chemistry of Life Processes Institute, Lurie Cancer Center. Northwestern University, Evanston, IL.
[4]Department of Chemical and Biological Engineering, Northwestern University, Evanston, IL.
[5]Department of Materials Science and Engineering, Northwestern University, Evanston, IL.
[6]Department of Biomedical Engineering, Northwestern University, Evanston, IL.
[7]Department of Microbiology-Immunology, Feinberg School of Medicine, Northwestern University, Chicago, IL.
[8]Department of Pharmacology, Feinberg School of Medicine, Northwestern University, Chicago, IL.
[9]Department of Chemistry and Biochemistry, University of California San Diego, La Jolla, CA.
[10]These authors contributed equally: Dauren Biyashev, Zofia E. Siwicka.
*Senior corresponding authors

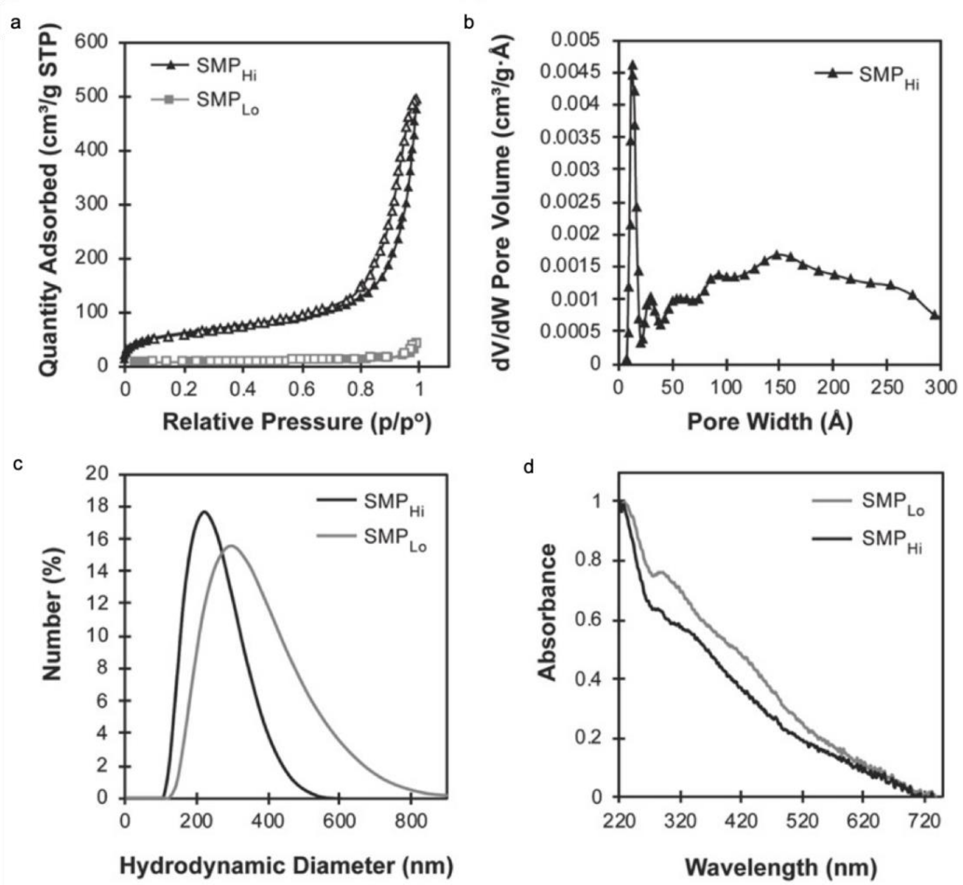

Supplemental Figure 1. High surface area Synthetic Melanin Particles (SMP$_{Hi}$) (black) and Low surface area Synthetic Melanin Particles (SMP$_{Lo}$) (gray) characterization. a) N_2 adsorption (solid markers) and desorption (open markers). **b)** Pore size distribution of SMP$_{Hi}$ determined using density functional theory (DFT). **c)** Dynamic light scattering. **d)** Ultraviolet visible spectroscopy.

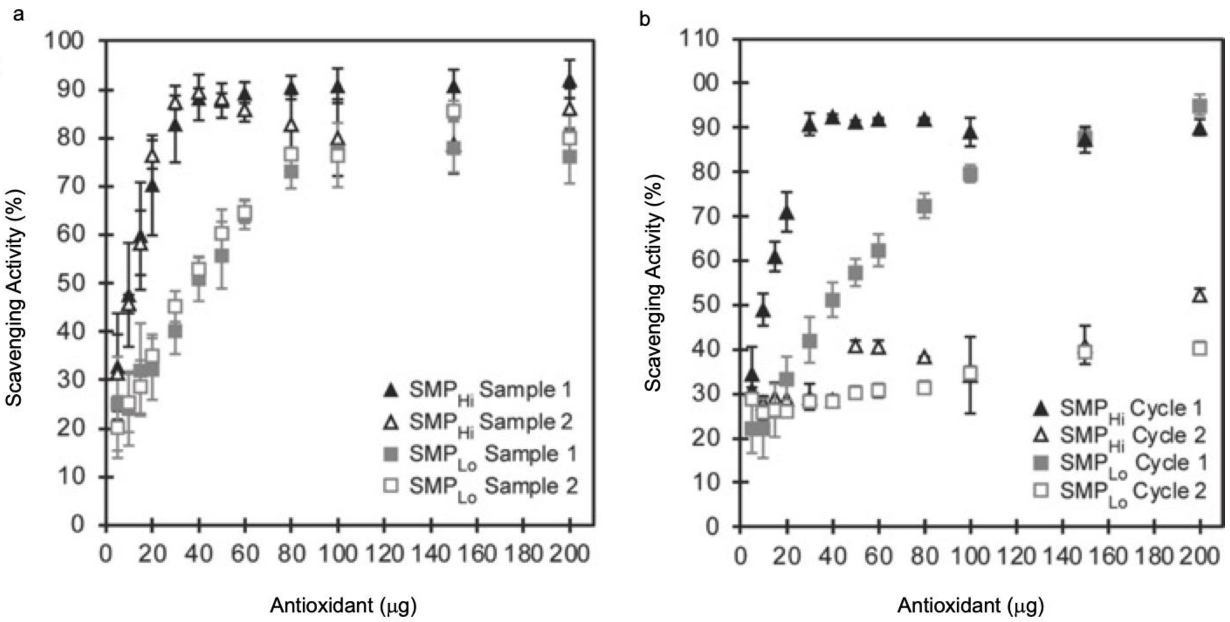

Supplemental Figure 2. DPPH radical scavenging activity of antioxidants of high surface area Synthetic Melanin Particles (SMP_{Hi}) (black triangle) and low surface area Synthetic Melanin Particles (SMP_{Lo}) (gray square). a) Scavenging activity for multiple batches. b**)** Scavenging activity cycles. DPPH assay was performed on particles (cycle 1) and washed with water after. Particles were left in water for one week and DPPH assay was performed again (cycle 2). Data are mean ± s.d.

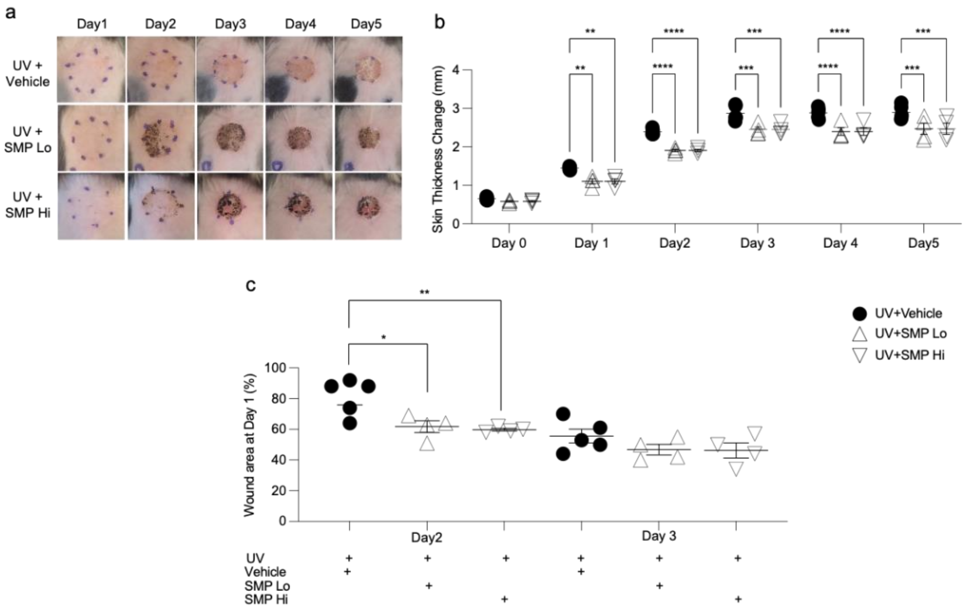

Supplemental Figure 3. SMP intervention improves skin healing after UV-induced injury. a) Representative images of the wounds on days 1 through 5. **b)** Bi-fold skin thickness measurements. Asterisks indicate statistical significance between NM+V and NM+SMP$_{Hi}$ groups. One-way ANOVA test used, n = 4-5 mice per group; *p<0.05, ***p<0.001, ****p<0.0001. **c)** Wound area reduction. n = 4-5 mice per group. One way ANOVA test, *p<0.05; **p<0.01. Data are mean ± s.e.m.

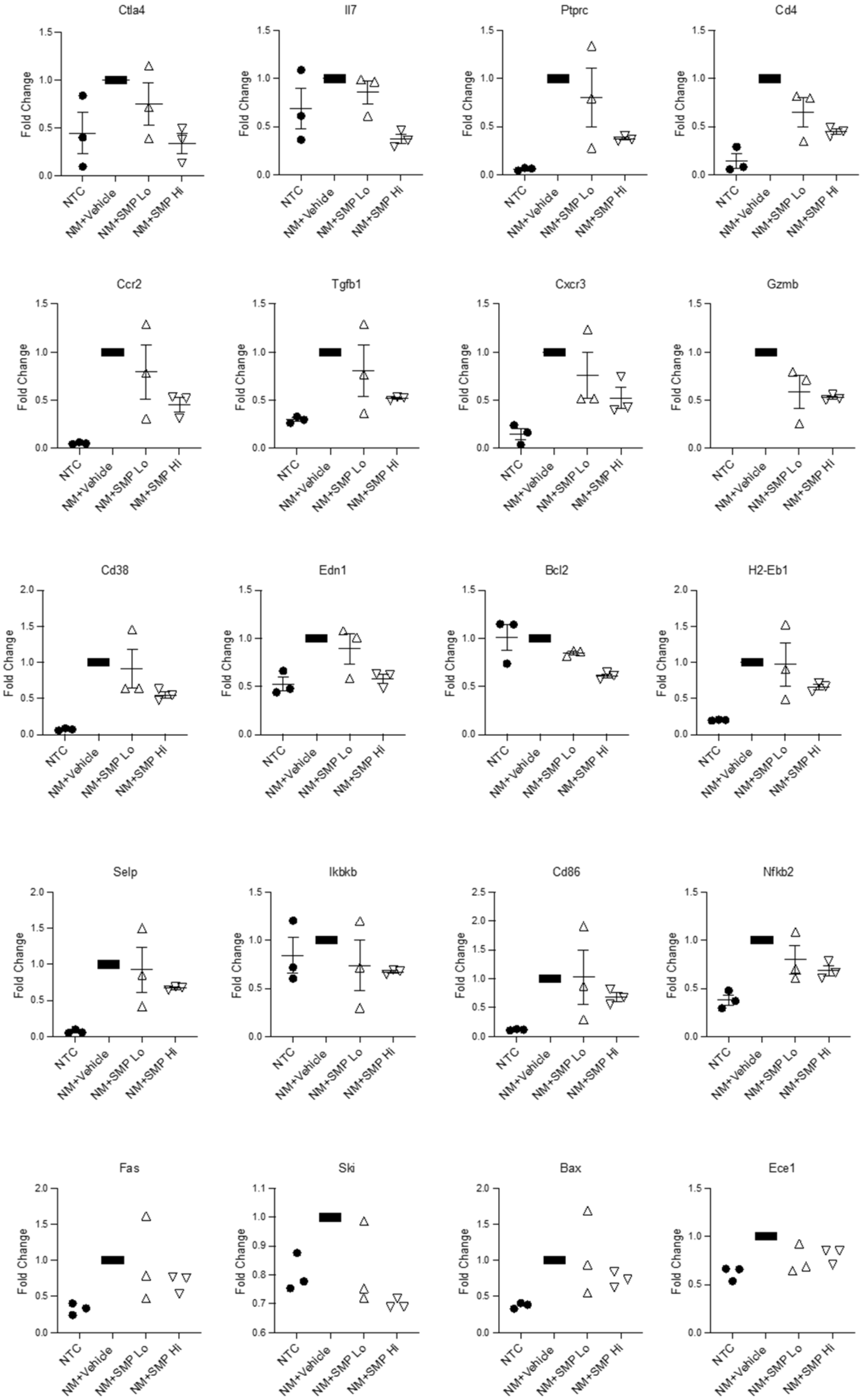

Supplemental Figure 4. TaqMan mouse immune array results. Genes significantly downregulated in the NM+SMP_{hi}-treated group (one sample t-test, all $p \leq 0.05$). The NM+Vehicle group was used as a reference. *Gzmb* values in the non-treated group were below detection level. n = 3-9 mice per group. Data are mean ± s.e.m.

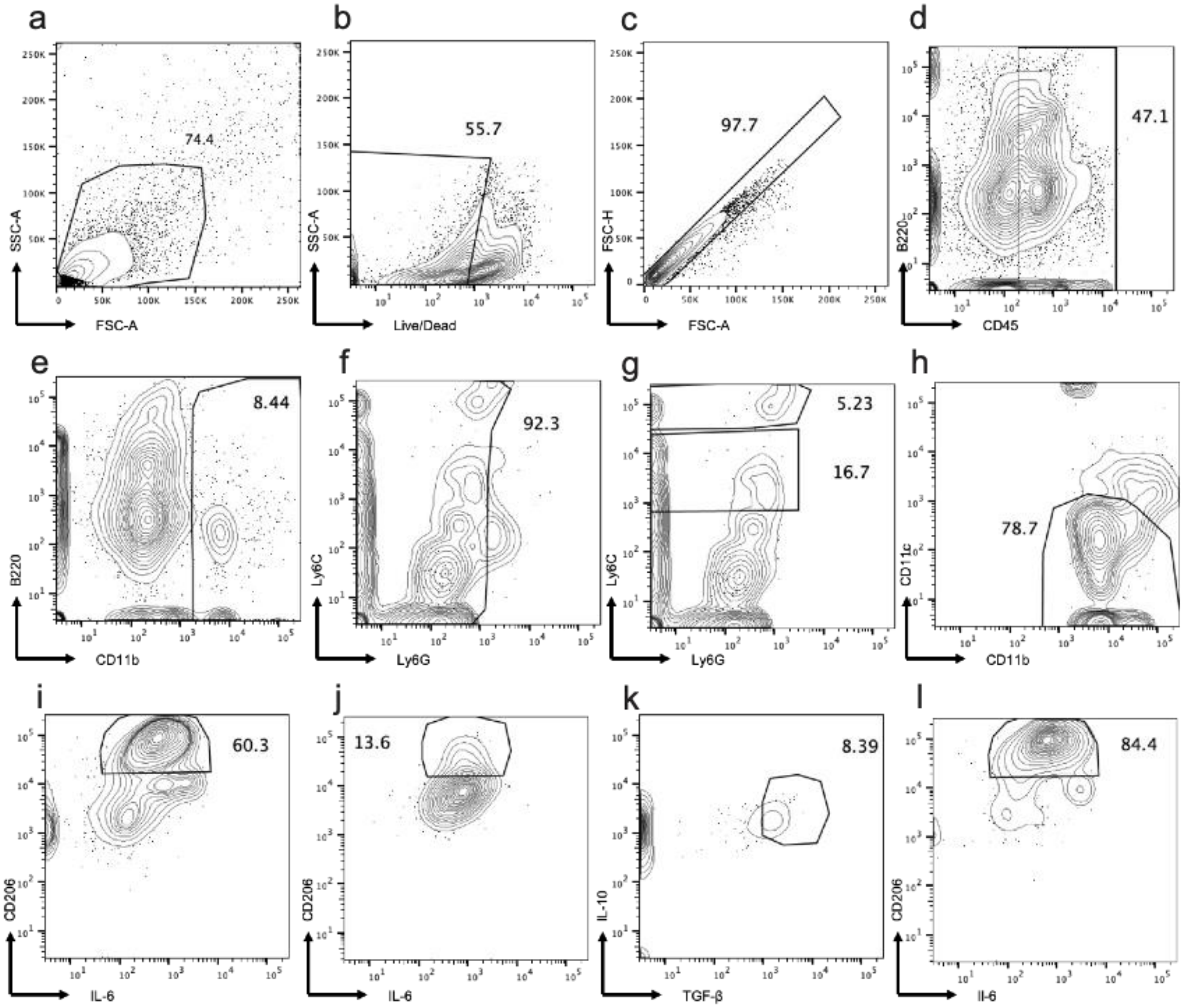

Supplemental Figure 5. Gating strategy for the APCs in cutaneous wound and spleen. The macrophage, inflammatory monocyte, and non-inflammatory monocyte were sequentially gated through panel **a-f**. Inflammatory monocytes and non-inflammatory monocytes are in the top and bottom gates in panel **g**, respectively. Panel **h** gate shows macrophages. Panels **i, j,** and **l** show the CD206hi macrophage, inflammatory monocytes, and non-inflammatory monocytes. Panel **k** indicates IL-10+TGF-β+ inflammatory monocytes.

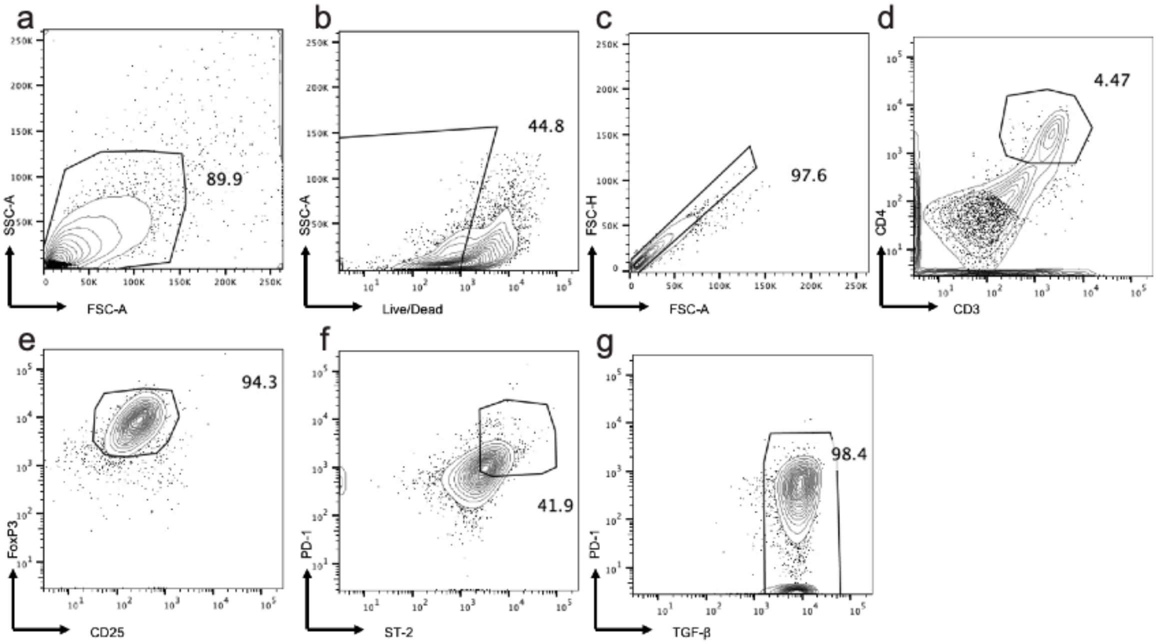

Supplemental Figure 6. Gating strategy for the T cell panel in cutaneous wound and spleen. CD4+ T cells were gated through panel **a** to **d**. Regulatory CD4+ T cells were subsequently gated through panel **e**. Panel **f** and **g** show PD-1+ST-2+ Treg and TGF-β+ Treg, respectively.

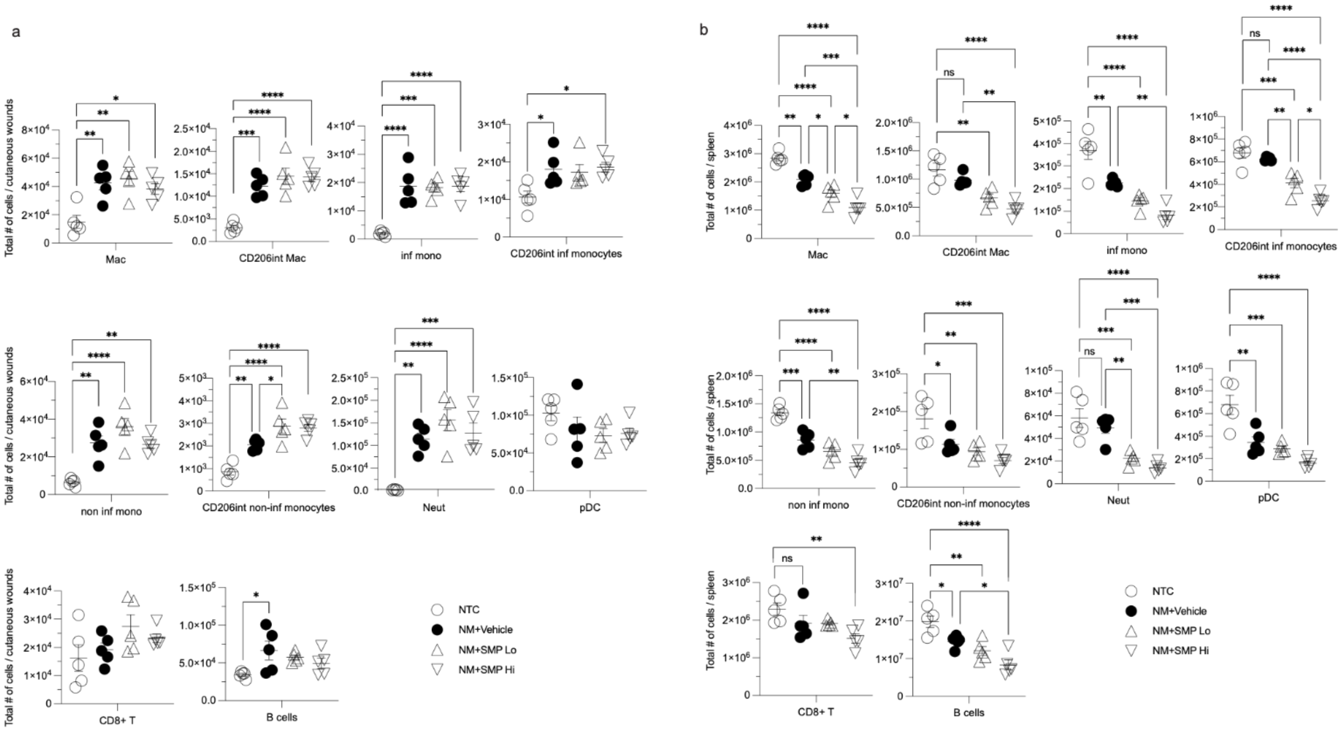

Supplemental Figure 7. SMP particles significantly modulate the traffic of inflammatory cells following cutaneous wound formation. Panel **a** shows APC subsets and lymphatic cell changes in skin, while Panel **b** shows those changes in spleen. *Tregs are CD4+CD25+FoxP3+ Tregs. One-way ANOVA was used for statistical analysis. N=4-5. *p<0.05, **p<0.01, ***p<0.001, ****p<0.0001. Data are mean ± s.e.m.

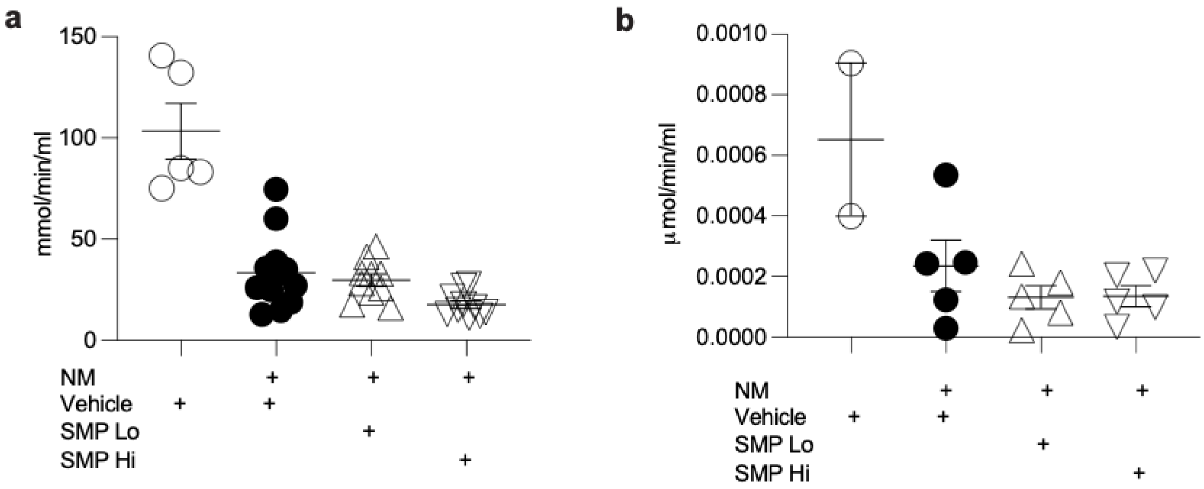

Supplemental Figure 8. SMP intervention did not change activities of catalase and thioredoxin reductase after nitrogen-mustard (NM) injury. **a)** Catalase activity. b**)** Thioredoxin reductase activity. Data are mean ± s.e.m.

Ethnicity (self-described)	Number of participants
African American	2
Caucasian	5
Hispanic, Latino or Spanish origin	2
Unspecified/Declined	1

Supplemental Table 1. Demographic information.

10 participants were all females, age range 25-51 y.o., median age±st.dev. 36.±6.4 years

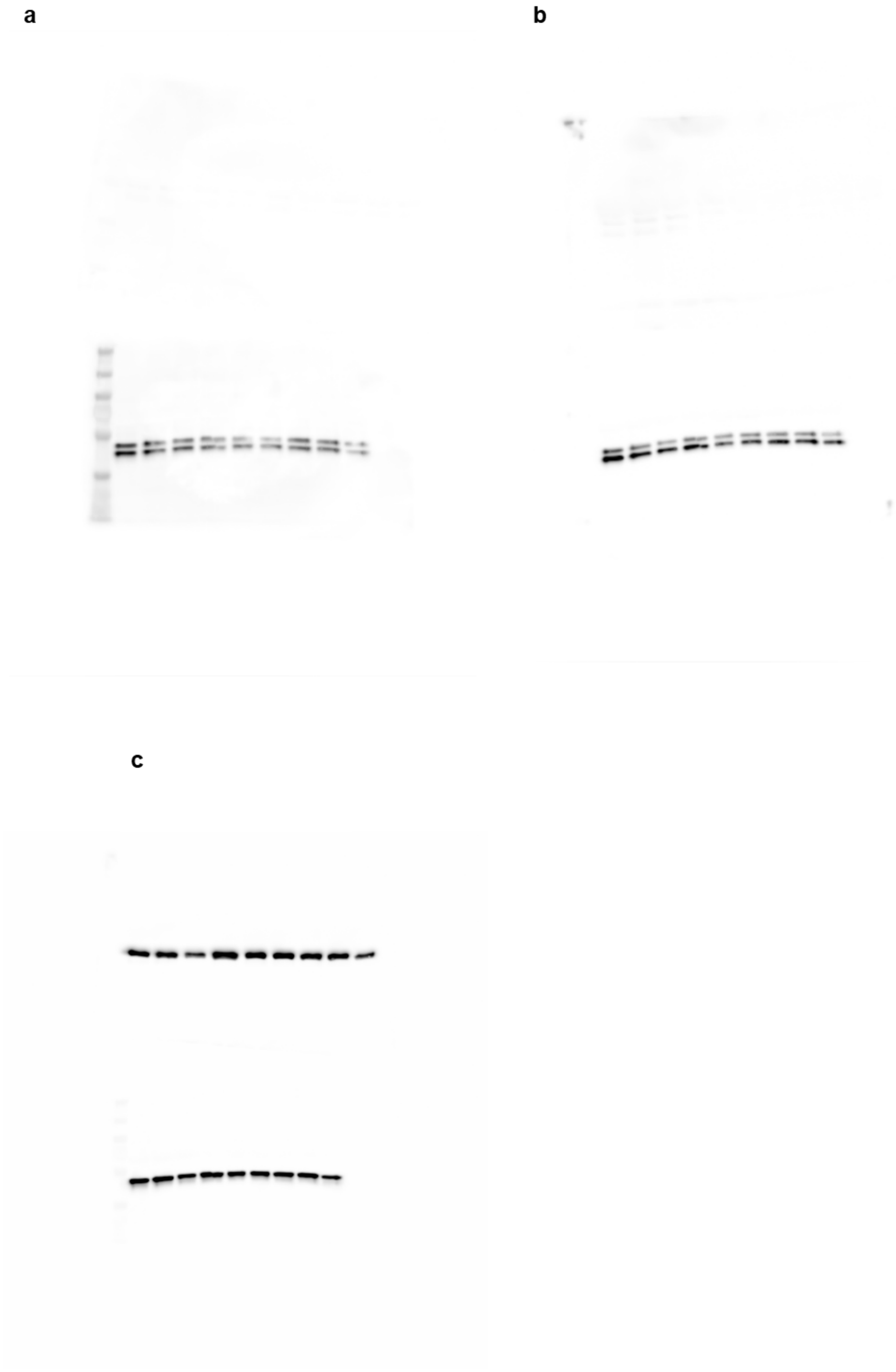

Supplemental Figure 9. a) Western blot of phospho ERK1/2 (lower membrane); b) Western blot of total ERK1/2 (lower membrane); c) Western blot of actin (lower membrane). Lane 1: protein standards ladder; lanes2-4: NM+vehicle; lanes 5-7: NM+SMP$_{Lo}$; lanes 8-10: NM+SMP$_{Hi}$
